# Node Centrality Measures Identify Relevant Structural MRI Features of Subjects with Autism

**DOI:** 10.3390/brainsci11040498

**Published:** 2021-04-14

**Authors:** Marcello Zanghieri, Giulia Menichetti, Alessandra Retico, Sara Calderoni, Gastone Castellani, Daniel Remondini

**Affiliations:** 1 Department of Electrical, Electronic, and Information Engineering “Guglielmo Marconi”, University of Bologna, I-40136 Bologna, Italy; marcello.zanghieri2@unibo.it; 2 Center for Complex Network Research, Department of Physics, Northeastern University, Boston, MA 02115, USA; g.menichetti@northeastern.edu; 3 Department of Medicine, Brigham and Women’s Hospital, Harvard Medical School, Boston, MA 02115, USA; 4 National Institute for Nuclear Physics (INFN), Pisa Division, I-56127 Pisa, Italy; alessandra.retico@pi.infn.it; 5 Department of Developmental Neuroscience, IRCCS Stella Maris Foundation, I-56128 Pisa, Italy; sara.calderoni@fsm.unipi.it; 6 Department of Clinical and Experimental Medicine, University of Pisa, I-56126 Pisa, Italy; 7 National Institute for Nuclear Physics (INFN), Bologna Division, I-40127 Bologna, Italy; 8 Department of Experimental, Diagnostic and Specialty Medicine, University of Bologna, I-40138 Bologna, Italy; 9 Department of Physics and Astronomy “Augusto Righi”, University of Bologna, I-40127 Bologna, Italy

**Keywords:** autism disorder, network theory, brain features

## Abstract

Autism spectrum disorders (ASDs) are a heterogeneous group of neurodevelopmental conditions characterized by impairments in social interaction and communication and restricted patterns of behavior, interests, and activities. Although the etiopathogenesis of idiopathic ASD has not been fully elucidated, compelling evidence suggests an interaction between genetic liability and environmental factors in producing early alterations of structural and functional brain development that are detectable by magnetic resonance imaging (MRI) at the group level. This work shows the results of a network-based approach to characterize not only variations in the values of the extracted features but also in their mutual relationships that might reflect underlying brain structural differences between autistic subjects and healthy controls. We applied a network-based analysis on sMRI data from the Autism Brain Imaging Data Exchange I (ABIDE-I) database, containing 419 features extracted with FreeSurfer software. Two networks were generated: one from subjects with autistic disorder (AUT) (DSM-IV-TR), and one from typically developing controls (TD), adopting a subsampling strategy to overcome class imbalance (235 AUT, 418 TD). We compared the distribution of several node centrality measures and observed significant inter-class differences in averaged centralities. Moreover, a single-node analysis allowed us to identify the most relevant features that distinguished the groups.

## 1. Introduction

According to the fifth edition of the Diagnostic and Statistical Manual of Mental Disorders (DSM-5) [1], autism spectrum disorders (ASDs) encompass a heterogeneous category of neurodevelopmental conditions characterized by a different level of symptom severity in two core domains: impairments in social communication and interaction along with restricted repetitive behaviors. In the previous version of the Diagnostic and Statistical Manual of Mental Disorders (DSM-IV-TR; [2]), the diagnostic category “Pervasive Developmental Disorders” (PDD) included distinct but phenotypically related categorical disorders: autistic disorder, Asperger’s Disorder, and pervasive developmental disorder-not otherwise specified (PDD-NOS), as well as two regressive neurodevelopmental disorders of early childhood characterized by autistic features: Rett’s syndrome, and childhood disintegrative disorder. Although the current elimination of diagnostic subtypes within autism spectrum disorder is motivated by the fact that the reliability of the diagnoses of different subtypes was poor across clinicians and unstable over time [3], debate is ongoing about its validity [4]. In fact, the ASD category includes subjects that highly differ in their genetic underpinnings [5] and clinical presentation [6], and this heterogeneity in turn impacts the ability to detect differences between ASD and control subjects, as suggested by the decrease in effect size over time [7]. With respect to neurobiological substrates identified using structural magnetic resonance imaging (sMRI), some studies have detected different and specific brain alterations among the DSM-IV-TR subtypes of PDD [8,9]), whereas others identified similar neural underpinnings shared among PDD subgroups [10,11]. Recently, these different views were reconciled by a large study that analyzed brain imaging data from the Autism Brain Imaging Data Exchange (ABIDE) cohorts: specifically, both common and unique cortical brain areas among Asperger’s, PDD-NOS, and autistic subgroups were found [12].

Given these premises, in our investigation, we focused on subjects with autistic disorder; i.e. the subtype with the highest levels of concordance when diagnosed using either the DSM-IV or DSM-5 diagnostic criteria [13]. To highlight structural brain characteristics that distinguish subjects with autistic disorder (AUT) from typically-developing controls (TD), we implemented an analysis based on network reconstruction that takes into account the relations (i.e. correlation) between all pairs of features used in our analysis (corresponding to nodes in the network). Then, we calculated several node centrality measures and compared the two subject groups at whole-network and single-node levels to characterize global differences and the role of specific features in generating such differences.

This paper is structured as follows: first, we describe the data selection criteria and the preprocessing methods implemented for the sMRI data and for the extracted features; then, the network-based approach and its implementation are presented; finally, the results are shown and discussed.

## 2. Materials and Methods

### 2.1. ABIDE Dataset and Sample Selection Criteria

We analyzed the structural MRI data of autistic and control subjects collected within the Autism Brain Imaging Data Exchange (http://fcon_1000.projects.nitrc.org/indi/abide/, accessed on 1 October 2020) I (ABIDE-I) initiative [14]. The ABIDE-I data collection is publicly available and consists of structural and functional MRI scans of 1112 individuals (539 subjects with autism and 573 typical controls), with and age range of 7–64 years, acquired in 17 different medical centers.

In this study, we focused on a subgroup of the ABIDE-I dataset, consisting of right-handed males that belong either to the AUT or TD groups (M=653 subjects): $M_{\mathrm{AUT}}=235$ AUT and $M_{\mathrm{TD}}=418$ TD subjects.

This subset was checked for age bias between the groups, given that age is the only relevant non-anatomical feature reported for all subjects in the dataset. We performed pairwise comparisons between four groups: (i) all TD subjects, (ii) all AUT subjects, (iii) right-handed male TD subjects, and (iv) right-handed male AUT subjects. Comparisons were made using a two-tailed, two-sample Kolmogorov-Smirnov test. The results are reported and detailed in Section 3.1, Table 1, showing no age bias for any comparison. In the Supplementary Materials, we show the plots of the dataset labeled for age and sampling site, which appear to be quite well mixed with respect to these factors.

### 2.2. Extraction of Descriptive Features

**Figure 1:**
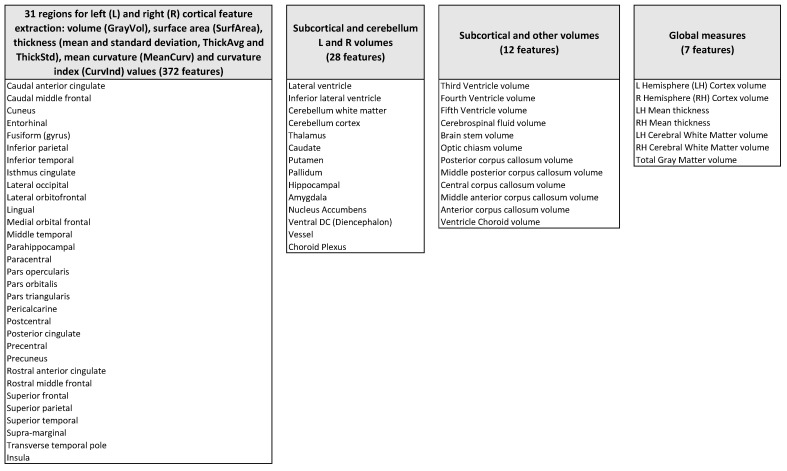
Scheme of the brain features extracted from the ABIDE-I sMRI data.

The structural MRI volumes were processed with the Freesurfer (https://surfer.nmr.mgh.harvard.edu, accessed on 1 October 2020) software (version 6.0) with the recon-all pipeline, (https://surfer.nmr.mgh.harvard.edu/fswiki/recon-all, accessed on 1 October 2020) which provides an automated segmentation of cortical and subcortical structures and generates a number of quantitative features for each brain region from the aseg.stats and h.aparc.stats output files, using asegstats2table and aparcstats2table Freesurfer scripts [15,16,17,18]. We considered 419 brain features (see the detailed list in Figure 1) extracted from structural MRI data defined according to the Desikan-Killiany-Tourville Atlas [19]:

- Volume, surface area, thickness (mean and standard deviation), mean curvature, and curvature index of 31 bilateral cortical structures (372 features);
- Volumes of bilateral sub-cortical structures and cerebellum (28 features);
- Volumes of other subcortical structures, including the corpus callosum and brainstem (12 features);
- Global measures–i.e. bilateral average global cortex volumes and thicknesses, bilateral white matter average volumes, and total gray matter volume (seven features).

The features were normalized to account for inter-individual variability according to the following criteria:

- Volumetric features were divided by the global brain volume, considering the FreeSurfer feature corresponding to brain segmented volume without ventricles (BrainSegVolNotVent);
- Cortical thicknesses were divided by the mean cortical thickness (computed as the average between the left and right hemisphere average thickness values);
- Cortical areas were divided by the sum of surface area values of all cortical structures.

We added additional features for each pair of features extracted from left and right homologous brain regions: the average and asymmetry (i.e., half difference) between left and right regions were computed and added to the initial set of features, yielding a total of N=818 features.

### 2.3. Weighted Correlation Networks (WCN)

We generated two groups of binarized and weighted correlation networks (WCNs) for TD ($G_{\mathrm{TD}}$) and AUT groups ($G_{\mathrm{AUT}}$), respectively. The analyses presented in the Section 3 refer to the comparison between a group of networks obtained from TD undersampling versus a single instance of the AUT network obtained by considering all AUT samples at once (see subsections below for details). In the Supplementary Materials (Section S3), we show the analogous results obtained by generating two sets of subsampled networks for both TD and AUT groups.

The networks were built as follows:

1. Each feature was identified as a node: $N_{\mathrm{node}}=N_{\mathrm{feat}}=419$;
2. For each group, the Spearman’s correlation matrix $r_{S}$ was computed;
3. Node pairs with $r_{S}$ exceeding the 0.85-th quantile of the whole Spearman’s coefficients distribution were considered as linked (self-loops were discarded);
4. After thresholding, we generated both a binarized and a weighted version of each network, with the weights assigned via the formula $w_{ii}=e^{-S_{ij}^{2}/\sigma^{2}}$, using the chordal distance $S_{ij}=\sin\left(\frac{1}{2}\arccos r_{S\;ij}\right)$ and setting σ=1.

In Step 2, the non-parametric Spearman correlation was chosen instead of Pearson linear correlation for two reasons: (i) it is more robust against high skewness and strong outliers, and (ii) it is more suitable for discrete-valued variables (as is the case for some features). In Step 3, we used a nonparametric threshold based on distribution percentiles to have a fixed link density (i.e., the number of links divided by the total possible links) for all networks: thresholding based on the 85th percentile means fixing the link density to ρ=0.15. This value was chosen empirically to prevent the formation of minor disconnected components or isolated nodes.

It is also worth remarking that the described pipeline produces results that are independent of any monotonic manipulation of the initial data (such as power or log-transformation, or normalizations), since all analyses are built upon the Spearman’s correlation based on rank.

### 2.4. Network Matrices and Node Centrality Measures

For each instance of the Spearman’s correlation matrix (obtained by patient subsampling; see Section 2.5) we generated a topological (i.e. binary) network *A* and a weighted network *W* of the same size, with nodes corresponding to brain features.

We calculated several node centrality measures specific for topological and weighted networks:

- The node degree $K_{i}$ and node strength $S_{i}$, defined as
  (1) $$K_{i}=\sum_{j=1}^{N}A_{ij};\;\;\;S_{i}=\sum_{j=1}^{N}W_{ij};$$
- The average degree and strength of each node’s nearest neighbors, denoted $K_{\mathrm{nn}i}$ and $S_{\mathrm{nn}i}$, respectively;
- The closeness centrality ${\mathrm{CC}}_{i}$ and weighted closeness centrality ${\mathrm{WCC}}_{i}$, defined as
  (2) $${\mathrm{CC}}_{i}=\frac{N-1}{\sum_{j=1}^{N}d_{\mathrm{topo}ij}};\;\;{\mathrm{WCC}}_{i}=\frac{N-1}{\sum_{j=1}^{N}d_{\mathrm{weighted}ij}}$$
  where $d_{\mathrm{topo}ij}$ and $d_{\mathrm{weighted}ij}$ are the topological and weighted distance between nodes *i* and *j*, respectively;
- The betweenness centrality ${\mathrm{BC}}_{i}$ and weighted betweenness centrality ${\mathrm{WBC}}_{i}$, defined as
  (3) BCi=1N−12∑j1≠ij2≠iσtopoj1j2(i)σtopoj1j2;WBCi=1N−12∑j1≠ij2≠iσweightedj1j2(i)σweightedj1j2
  where, for both the topological and the weighted network, $\sigma_{j_{1}j_{2}}$ is the number of shortest paths between nodes *i* and *j*, and $\sigma_{j_{1}j_{2}}\left(i\right)$ is the number of paths passing through node *i*;
- The clustering coefficient ${\mathrm{Clust}}_{i}$, defined as the fraction of pairs of a node’s adjacent nodes that are also adjacent to each other (and thus not defined for *i* s.t. $K_{i}<2$), computed as
  (4) Clusti=(A3)ii/2Ki2;
- *Spectral Centrality*${\mathrm{SC}}_{i}$ and *Weighted Spectral Centrality*${\mathrm{WSC}}_{i}$, defined as:
  (5) $$\begin{array}{cc} {\mathrm{SC}}_{i}^{\left(1\right)} & =\sum_{j=1}^{N}A_{ij}{\left(\nu_{i}^{\mathrm{topo}}-\nu_{j}^{\mathrm{topo}}\right)}^{2} \end{array}$$
  (6) $$\begin{array}{cc} {\mathrm{WSC}}_{i}^{\left(1\right)} & =\sum_{j=1}^{N}W_{ij}{\left(\nu_{i}^{\mathrm{weighted}}-\nu_{j}^{\mathrm{weighted}}\right)}^{2} \end{array}$$
  where the superscript “(1)” is used to specify that the 1st spectral centrality is considered, and $v_{1}^{\mathrm{topo}}=\left(\nu_{1}^{\mathrm{topo}},\cdots ,\nu_{N}^{\mathrm{topo}}\right)$ and $v_{1}^{\mathrm{weights}}=\left(\nu_{1}^{\mathrm{weights}},\cdots ,\nu_{N}^{\mathrm{weights}}\right)$ denote the first nontrivial eigenvector of the Laplacian for the topological and the weighted networks, respectively [20].

### 2.5. Network Subsampling

To compare the values of the considered centrality measures between TD and AUT groups, dataset imbalance is a critical issue, for two reasons:

- The large difference in samples in $G_{\mathrm{TD}}$ and $G_{\mathrm{AUT}}$ ($M_{\mathrm{TD}}=418$, $M_{\mathrm{AUT}}=235$) can cause a different amount of fluctuations in sample correlation $r_{S}$;
- By reconstructing a single network instance for each group, only two values would be produced (one for $G_{TD}$ and one for $G_{AUT}$), making a statistical comparison impossible between the two groups.

We chose to overcome these limitations by performing a subsampling with replacements on the $G_{\mathrm{TD}}$ subjects, which allowed us both (i) to work with networks built from an identical sample number $M_{\mathrm{sub}}<\min\left\{M_{\mathrm{TD}},M_{\mathrm{AUT}}\right\}=235$ and thus with an equal number of fluctuations and (ii) to obtain a distribution of network measures for the control group instead of a single set of values. The subsampling procedure was performed as follows: from the control group with $M_{\mathrm{TD}}=418$ subjects, we randomly extracted $M_{\mathrm{sub}}=235$ samples (corresponding to the size of $G_{\mathrm{AUT}}$) and for each extraction, we generated an instance of the control network. The extraction step was repeated $K=5\;\times \;10^{4}$ times, and each time all centrality measures were calculated.

Two different analyses were performed:

- Whole-network statistics: For each network, a single centrality value was calculated as the average over all node centrality measures (obtaining a distribution of $5\times 10^{4}$ values for each centrality measure in the TD group to be compared with a single value for the AUT group);
- Single-node statistics: We compared the centrality values separately for each node (thus obtaining a $818\times 5\times 10^{4}$ table of centrality values for the TD subsampled group, to be compared with the 818 values for the single instance of the AUT group).

All the analyses were performed with MATLAB R2019b on a 2.10GHz Intel Xeon CPU E5-2620 v4 ($K=5\times 10^{4}$ repetitions took about 8 h of computation).

### 2.6. Group Comparison

We compared the values of the $G_{\mathrm{AUT}}$ network with the distribution of corresponding values for $G_{\mathrm{TD}}$ networks (processed with Box-Cox transforms), using the *z*-score distance $z_{n}=\left(c_{n}^{\mathrm{AUT}}-{\langle c_{n}\rangle }^{\mathrm{TD}}\right)/\sigma_{n}$, where the *n* index ranges through all centrality measures, $c^{\mathrm{AUT}}$ is the value of the single AUT instance, ${\langle c_{n}\rangle }^{\mathrm{TD}})$ is the average over all TD subsampled network measures, and $\sigma_{n}$ is the standard deviation of $G_{\mathrm{TD}}$ values. The score was calculated for the whole network or singularly for each node, depending on the type of analysis described in the previous subsection. In the Supplementary Materials (Section S3), we also show the results of the comparison between a distribution of TD networks and a distribution of AUT networks obtained by a similar subsampling procedure, which are in complete agreement with the analysis shown in the paper.

## 3. Results

### 3.1. Age Bias in TD and AUT Groups

Four sample groups were pairwise compared (with a latin square design) with respect to age: all TD subjects, all AUT subjects, right-handed male TD subjects, and right-handed male AUT subjects. Statistical comparisons were conducted via two-tailed, two-sample Kolmogorov-Smirnov tests. The outcomes in terms of *p*-values are reported in Table 1. No comparison showed a statistically significant difference; thus, the choice to restrict our analysis to right-handed males did not introduce any age-related bias as compared to the full ABIDE dataset.

### 3.2. Distribution of the Feature Correlation Coefficients

**Figure 2:**
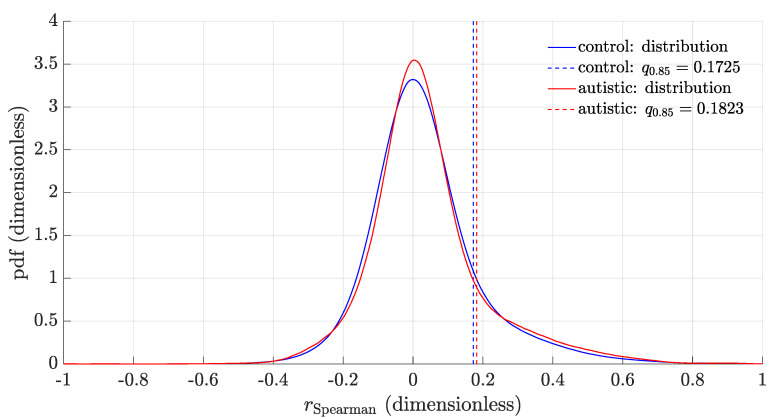
Distribution of the Spearman’s correlation coefficients for the TD and AUT groups. The 85th percentile of each distribution is also shown (average value for TD, a single value for AUT), used as the threshold for network reconstruction.

The distributions of the Spearman’s correlation coefficients $r_{S}$ for the TD and AUT groups are shown in Figure 2. For the AUT group, the distribution of the single network realization $G_{\mathrm{AUT}}$ is reported; for the control group, we instead report the average distribution of the $r_{S}$ coefficients over the $K=5\times 10^{4}$ subsampled networks. The two distributions are different, with the AUT values being more concentrated in the peak and in the tails as compared to TD. In particular, the AUT 85th quantile is higher than the 85th quantile for TD ($q_{\mathrm{TD}}=0.1725$ and $q_{\mathrm{AUT}}=0.1823$).

### 3.3. Correlation between Centrality Measures

**Figure 3:**
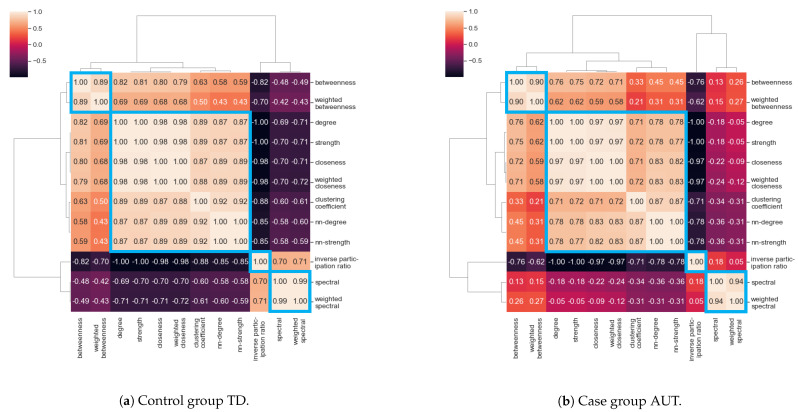
Spearman correlation-based cluster maps of the centrality measures.

Since different centrality measures can convey similar information, we inspected the Spearman’s correlation between the vectors of 818 values for all nodes in the networks, calculated separately for the TD and the AUT groups. For the TD group, averages were taken over the $K=5\times 10^{4}$ repetitions, obtaining one value per node. The correlations between the vectors of node centrality measures were used as distances to generate a cluster map as shown in Figure 3. We observe that some pairs of centrality measures have very high correlation values (>0.99). Even if the correlation values are different for TD and AUT groups, the resulting cluster structure is the same, highlighting four main clusters of centrality measures:

- Cluster I: Betweenness centrality and weighted betweenness centrality;
- Cluster II: Degree, strength, nearest-neighbor degree, nearest-neighbor strength, closeness centrality, weighted closeness centrality, clustering coefficient;
- Cluster III: Inverse participation ratio;
- Cluster IV: Spectral centrality and weighted spectral centrality.

This result suggested that we should consider one type of centrality measure for each cluster, since highly correlated centrality measures are supposed to provide the same information about node relevance.

### 3.4. Whole-Network Comparison

**Figure 4:**
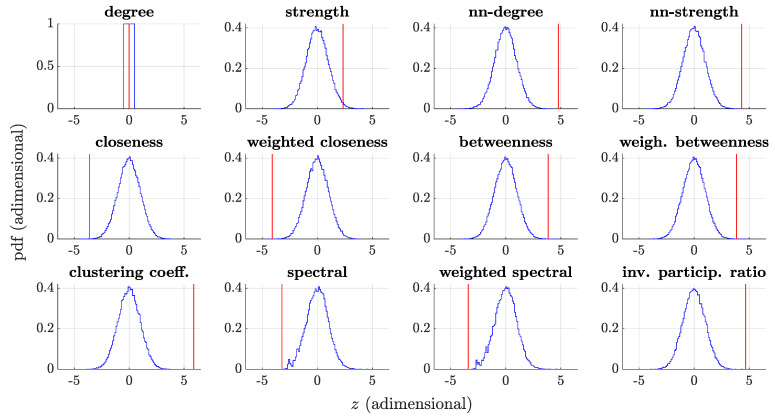
Box-Cox normalized distributions for the TD group (blue) and single values for the AUT group (red) for the node centrality measures.

In the comparison at the global network level, the centrality measures were averaged over all the nodes within each network, obtaining $K=5\;\times \;10^{4}$ values for each of the 12 centrality measures in the TD group, and 12 single values for the AUT group. The Box-Cox regularized distributions for TD group and the single values for AUT group are displayed in Figure 4 for each centrality measure. The statistical significance of the group differences was computed by counting how many values from each TD distribution were more extreme than the corresponding AUT value, yielding an empirical *p*-value for each centrality measure based on the percentile. The results are shown in Table 2, reporting both the *z*-scores and the *p*-values. In the table, the centrality measures are grouped based on the clusters identified in Section 3.3, and ranked within each cluster according to the *z*-score effect size. In general, we observe large differences between the TD distribution and AUT single values, reflecting the different structure of the two networks at a global level. The results of the analyses with subsampling for both AUT and TD groups are shown in the Supplementary Materials and are in good agreement with those shown here.

### 3.5. Single-Node Comparison

The same analysis was performed at a single-node level, comparing the TD distributions with the single AUT values. Ranking the z-score of the single nodes allowed us to identify which nodes—i.e., brain features—mostly contributed to the differences between TD and AUT groups. We only analyzed the centrality measures with the largest difference within each cluster: betweenness centrality (BC) for cluster I, clustering coefficient (Clust) for cluster II, inverse participation ratio (IPR) for cluster III, and weighted spectral centrality (WSC) for cluster IV. Table 3, Table 4, Table 5 and Table 6 report the top five brain features for the chosen centrality measures. We comment on the biological relevance of these identified brain features in Section 4.

### 3.6. Network Visualization

**Figure 5:**
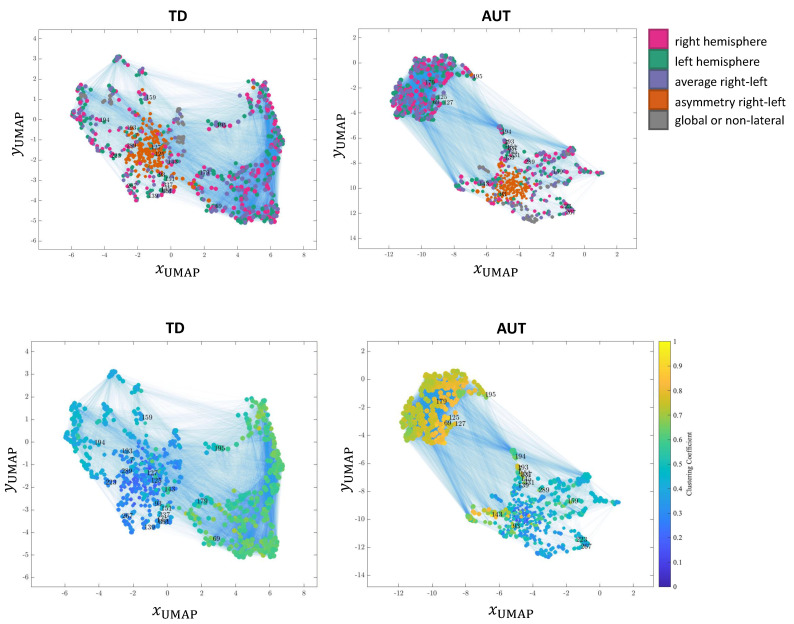
Representation of TD and AUT weighted networks with UMAP layout. **Top**: nodes colored by feature laterality. Features refer to the right or left hemisphere, to the average or to the difference (asymmetry) between corresponding left-right features, or global features referring to the whole brain. **Bottom**: nodes colored by clustering coefficient values. We annotate the number IDs of the top-20 features with the largest variation in clustering coefficients.

UMAP embedding [21] was used to visualize the networks (Figure 5), where for the TD network one of the random subsamplings was represented. In the top row, nodes are colored according to feature laterality; i.e. if they are global features for the whole brain, if they belong to one hemisphere, or if they are a combination of both hemisphere features (as described above). In the bottom row, the color scale is based on clustering coefficient values and the centrality measure with the highest difference between TD and AUT networks.

Two observations emerge in terms of the global structure, even if both network have an identical link density: first, the AUT network presents more segregated clusters as compared to the TD network; secondly, in the AUT network, features tend to form clusters with similar values of clustering coefficients.

## 4. Discussion

In the last decade, much progress has been made in uncovering neuroanatomical underpinnings that differentiate ASD patients from TD at the group level (see [22] for a recent review), but most neuroimaging studies have been conducted with small sample sizes that in turn could produce inconsistent and poorly replicated findings [23].

To try to overcome biases related to underpowered studies [24], worldwide data-sharing initiatives have been developed also in the ASD field, including ENIGMA ASD (http://enigma.ini.usc.edu/ongoing/enigma-asd-working-group/, accessed on 1 October 2020) working group, National Database for Autism Research (NDAR) (https://ndar.nih.gov/, accessed on 1 October 2020), and Autism Brain Imaging Data Exchange (ABIDE) [14].

In the current study, we have applied second-order statistics to brain features embedded in a network framework to identify differences in their interrelations in the TD and AUT groups. Node centrality measures then allowed us to rank and identify the single features that were most associated with the differences found between TD and AUT patients.

In particular, the structure of frontal brain regions seems to mostly differentiate ASD from TD individuals, especially in the inferior frontal cortex, orbitofrontal cortex, and middle frontal cortex. In particular, we identified a significant alteration of the pars triangularis belonging to Broca’s area–a region of inferior frontal cortex implicated in higher-order skills that are generally impaired in ASD individuals, including expressive language, action imitation, attribution of mental states, and empathy [25]. The medial orbitofrontal cortex is a critical brain region for regulating social behavior and inhibiting inappropriate social conduct [26] and has been also implicated in repetitive behavior–a core symptom of ASD [27]. Rostral middle frontal region (RMFG), a sub-region of the dorsolateral prefrontal cortex (DLPFC), is critical for executive functions, the cognitive processes that allow the selection of actions appropriate to our current activities or goals [28], among which dysfunction in emotion regulation [29], planning [30], mental flexibility [31], response inhibition [32], working memory [33], and cognitive control [34] have been consistently identified in ASD. The caudal middle frontal gyrus (MFG) (encompassed in the DLPFC) is also implicated in higher-level cognition: specifically, a hierarchical model has been proposed according to which caudal MFC provides cognitive control over current processing (e.g., stimuli translated into actions), while RMFC provides cognitive control over future processing (e.g., goals or plans). The superior frontal gyrus (SFG) belongs to the anterior rostral region of the medial frontal cortex (arMFC) and represents an important node of the social brain that has a role in social cognitive tasks requiring theory of mind (ToM) abilities [35]. The middle temporal gyrus (MTG) is part of the network that is alleged to be the basis of language, emotion, and social cognition, and has also been proposed to underpin ASD deficits in mentalizing, set-shifting, irony processing, and eye-gaze processing [36]. The anterior-most region of the parahippocampal gyrus, the entorhinal cortex, is part of the key anatomical structures critical for memory function, which is disrupted in ASD subjects when evaluated with a comprehensive test battery [37,38]. Importantly, the altered cortical thickness we observed in the entorhinal cortex is consistent with findings derived from the large ASD data set of the ENIGMA-ASD working group [39]. The precentral gyrus is part of the core human mirror neuron system–a neural circuit that processes information related to the perception and execution of biological motions, whose dysfunction has been related to a set of ASD symptoms (impairment in communication, language, and the capacity to understand others) [40].

Besides the well-replicated alterations in the frontal cortical regions [41,42]), structural alterations in other brain structures were detected in the current work. For example, functional differences in the superior parietal lobe (SPL) have previously been linked to impairments in motor learning [43], but also to social symptoms in ASD [44]. The precuneus operates together with the paracentral lobule, which is part of the SPL, to produce a sense of self and of spatial environment [45] and to modulate social interactions [46]. Interestingly, a brain system in the precuneus/SPL region with reduced functional connectivity has been described using the ABIDE data set [14] in ASD subjects [47] and has been related to the impaired representation of oneself in the world, which in turn may impact on the theory of mind skills of ASD patients [48]. The involvement of the insula has been previously observed in the ASD literature, in terms of hypoactivation during socio-emotional processing tasks [49] and hyperactivation in response to executive function processes in early developmental stages [50]. Conversely, the largest multi-site MRI brain morphometry study to date that included, inter alia, the ABIDE I [14] and ABIDE II [51] datasets (N=1571 for ASD, N=1651 for TD) did not detect differences in insula volume or thickness between individuals with ASD and typical controls [39]. The insula plays a crucial role in the “salience network”, which is charged with integrating external stimuli with self-perceptions and emotional states, alterations of which are related to several impairments of ASD [52].

We found structural alterations also in the postcentral gyrus (PCG) in ASD children compared with TD. The human PCG includes the primary somatosensory cortex, which is critical for somatosensory information processing (touch, proprioception, nociception, and temperature). Crucially, hypo and hyper-reactivity to sensory stimuli are distinguishing features of ASD that are included within the main ASD criteria of restricted interests and repetitive behaviors in the DSM-5 and may negatively impact the daily life of ASD individuals and their families [53]. The atypical brain structure we detected in PCG is also consistent with previous studies in ASD subjects [54,55]. Besides this, we identified brain alterations in the lateral occipital region, which plays a specific role in object recognition [56]. Even if object recognition is not specifically impaired in ASD, as in the case of face recognition [57], it is characterized by an atypical time course (i.e., delay in the processing of global information) compared with TD peers [58].

In summary, the current study, implementing a network-based analysis of sMRI data, suggests that neural underpinnings of ASD include GM alterations in distributed cortical and subcortical regions crucial for socio-cognitive and/or motor processes.

The second-order relations between features (i.e., their correlation) we extracted with our network approach highlight relationships between the levels of the features rather than differences in the feature values between AUT and TD groups. This might suggest that signatures for patient classification, and possibly for their stratification related to different levels of severity in the pathology, might be sought in a combination of ratios between the values of some specific features (e.g., the most relevant we identified and some of their closest neighboring features) that could be measured at a single-patient level. This analysis goes beyond the scope of this paper and requires progress towards new studies that would require at least another patient database in order not to be biased by the results obtained with ABIDE I dataset and a sufficient sample size for the scopes of classification and stratification.

## 5. Conclusions

In this work, we have considered a well-known dataset related to autistic disorder, namely ABIDE I, which is one of the largest publicly available, and have performed a network-based analysis of the features extracted from the MR images available for the patients in order to characterize their second-order relationships (i.e., correlations between variables, transformed in weighted networks for control and autistic patient groups).

The analysis showed significant differences both at a global level (properties of the whole network averaged over single-node measures) and also at the level of single features. With this latter analysis, we could rank features based on their statistical score, and we could provide a biological interpretation of these features related to the known literature and to specific brain areas and cognitive functions. 

## Figures and Tables

**Table 1:** *p*-values from the Kolmogorov-Smirnov tests ($p_{\mathrm{KS}}$) used to compare group age. TD: typically developing; AUT: autistic disorder.

*p* ${}_{\mathrm{KS}}$	all TD	all AUT	Right-Handed Male TD	Right-Handed Male AUT
**all TD**	1	0.699	0.698	0.553
**all AUT**		1	0.408	0.993
**right-handed male TD**			1	0.784
**right-handed male AUT**				1

**Table 2:** The *z*-scores and *p*-values for the node centrality measures, grouped according to the clusters found in Section 3.3 and ranked within each cluster based on the *z*-score.

Cluster	Centrality Measure	*z*-Score	*p*-Value
I	Betweenness	3.85	$6\times 10^{-5}$
	Weighted betweenness	3.84	$6\times 10^{-5}$
II	Clustering coefficient	5.88	<$2\times 10^{-5}$
	nn-degree	4.77	<$2\times 10^{-5}$
	nn-strength	4.31	<$2\times 10^{-5}$
	Weighted closeness	−4.09	$6\times 10^{-5}$
	Closeness	−3.62	0.00014
	Strength	2.33	0.011
	Degree	0 by constr.	1 by constr.
III	Inverse participation ratio	4.69	<$2\times 10^{-5}$
IV	Weighted spectral	−3.40	0.0019
	Spectral	−3.21	0.0019

**Table 3:** Top-ranking brain features of betweenness centrality (Cluster I).

Rank	Hemisphere	Brain Area	Measure	z_BC_
1	R	Postcentral	Gray volume	2.808
2	R	Medial orbitofrontal	Gray volume	2.584
3	R	Superior frontal	Average thickness	2.310
4	R	Rostral middle frontal	Average thickness	2.214
5	L	Rostral middle frontal	Gray volume	2.207

**Table 4:** Top-ranking brain features of clustering coefficient (Cluster II).

Rank	Hemisphere	Brain Area	Measure	z_Clust_
1	R	Pars triangularis	Surface area	6.705
2	R	Caudal middle frontal	Surface area	6.046
3	R	Rostral middle frontal	Surface area	5.893
4	R	Caudal middle frontal	Gray volume	5.686
5	R	Postcentral	Mean curvature	5.097

**Table 5:** Top-ranking brain features of inverse participation ratio (IPR) (Cluster III).

Rank	Hemisphere	Brain Area	Measure	z_IPR_
1	R	Middle temporal	Surface area	4.087
2	R	Entorhinal	Standard thickness	3.643
3	L	Lateral occipital	Gray volume	2.772
4	L	Insula	Surface area	2.735
5	R	Insula	Surface area	2.734

**Table 6:** Top-ranking brain features of weighted spectral centrality (WSC) (Cluster IV).

Rank	Hemisphere	Brain Area	Measure	z_WSC_
1	L	Precentral	Mean curvature	−1.836
2	L	Precuneus	Standard thickness	−1.708
3	L	Postcentral	Curvature index	−1.705
4	L	Superior parietal	Curvature index	−1.679
5	L	Superior frontal	Standard thickness	−1.626

## Data Availability

Original data used in this study are publicly available within the ABIDE-I initiative http://fcon_1000.projects.nitrc.org/indi/abide/, accessed on 1 October 2020). Preprocessed data are available from the authors upon request.

## Supplementary Materials

The following supplementary file is available at https://www.mdpi.com/article/10.3390/brainsci11040498/s1. Part S1: Stratification with respect to age and multi-site data collection, Part S2: Single feature statistical analysis, Part S3: Results with resampling for both TD and AUT.

## Abbreviations

The following abbreviations are used in this manuscript:

TD	Typically-developing subjects
AUT	Autistic disorder/autistic subjects
*M*	Number of subjects
*N*	Number of features/nodes (equivalent)
$r_{S}$	Spearman correlation coefficient
*G*	Network (*G* standing for *graph*)
*A*	Adjacency matrix
*W*	Weights matrix
(topo)	Relative to the topological network
(weighted)	Relative to the weighted network
*K*	Degree
*S*	Strength
$K_{\mathrm{nn}}$	Average degree of nearest neighbors
$S_{\mathrm{nn}}$	Average strength of nearest neighbors
CC	Closeness centrality
WCC	Weighted closeness centrality
BC	Betweenness centrality
WBC	Weighted betweenness centrality
*C*, Clust	Clustering coefficient
SC	Spectral centrality
WSC	Weighted spectral centrality
IPR	Inverse participation ratio

## References

[1] American Psychiatric Association (2013). Diagnostic and Statistical Manual of Mental Disorders (DSM-5®).

[2] American Psychiatric Association (2000). Diagnostic and Statistical Manual of Mental Disorders-IV Text Revision.

[3] Lord C., Bishop S.L. (2015). Recent advances in autism research as reflected in DSM-5 criteria for autism spectrum disorder. Annu. Rev. Clin. Psychol..

[4] Mottron L., Bzdok D. (2020). Autism spectrum heterogeneity: Fact or artifact?. Mol. Psychiatry.

[5] Grove J., Ripke S., Als T.D., Mattheisen M., Walters R.K., Won H., Pallesen J., Agerbo E., Andreassen O.A., Anney R. (2019). Identification of common genetic risk variants for autism spectrum disorder. Nat. Genet..

[6] Green J. (2019). Editorial Perspective: Delivering autism intervention through development. J. Child Psychol. Psychiatry.

[7] Rødgaard E.M., Jensen K., Vergnes J.N., Soulières I., Mottron L. (2019). Temporal changes in effect sizes of studies comparing individuals with and without autism: A meta-analysis. JAMA Psychiatry.

[8] McAlonan G.M., Suckling J., Wong N., Cheung V., Lienenkaemper N., Cheung C., Chua S.E. (2008). Distinct patterns of grey matter abnormality in high-functioning autism and Asperger’s syndrome. J. Child Psychol. Psychiatry.

[9] Toal F., Daly E., Page L., Deeley Q., Hallahan B., Bloemen O., Cutter W., Brammer M., Curran S., Robertson D. (2010). Clinical and anatomical heterogeneity in autistic spectrum disorder: A structural MRI study. Psychol. Med..

[10] Kwon H., Ow A.W., Pedatella K.E., Lotspeich L.J., Reiss A.L. (2004). Voxel-based morphometry elucidates structural neuroanatomy of high-functioning autism and Asperger syndrome. Dev. Med. Child Neurol..

[11] Via E., Radua J., Cardoner N., Happé F., Mataix-Cols D. (2011). Meta-analysis of gray matter abnormalities in autism spectrum disorder: Should Asperger disorder be subsumed under a broader umbrella of autistic spectrum disorder?. Arch. Gen. Psychiatry.

[12] Qi S., Morris R., Turner J.A., Fu Z., Jiang R., Deramus T.P., Zhi D., Calhoun V.D., Sui J. (2020). Common and unique multimodal covarying patterns in autism spectrum disorder subtypes. Mol. Autism.

[13] Mazurek M.O., Lu F., Symecko H., Butter E., Bing N.M., Hundley R.J., Poulsen M., Kanne S.M., Macklin E.A., Handen B.L. (2017). A prospective study of the concordance of DSM-IV and DSM-5 diagnostic criteria for autism spectrum disorder. J. Autism Dev. Disord..

[14] Di Martino A., Yan C.G., Li Q., Denio E., Castellanos F.X., Alaerts K., Anderson J.S., Assaf M., Bookheimer S.Y., Dapretto M. (2014). The autism brain imaging data exchange: Towards a large-scale evaluation of the intrinsic brain architecture in autism. Mol. Psychiatry.

[15] Dale A.M., Fischl B., Sereno M.I. (1999). Cortical surface-based analysis: I. Segmentation and surface reconstruction. Neuroimage.

[16] Fischl B., Sereno M.I., Dale A.M. (1999). Cortical surface-based analysis: II. inflation, flattening, and a surface-based coordinate system. Neuroimage.

[17] Fischl B., Salat D.H., Busa E., Albert M., Dieterich M., Haselgrove C., Van Der Kouwe A., Killiany R., Kennedy D., Klaveness S. (2002). Whole brain segmentation: Automated labeling of neuroanatomical structures in the human brain. Neuron.

[18] Fischl B., Van Der Kouwe A., Destrieux C., Halgren E., Ségonne F., Salat D.H., Busa E., Seidman L.J., Goldstein J., Kennedy D. (2004). Automatically parcellating the human cerebral cortex. Cereb. Cortex.

[19] Desikan R.S., Ségonne F., Fischl B., Quinn B.T., Dickerson B.C., Blacker D., Buckner R.L., Dale A.M., Maguire R.P., Hyman B.T. (2006). An automated labeling system for subdividing the human cerebral cortex on MRI scans into gyral based regions of interest. Neuroimage.

[20] Pauls S.D., Remondini D. (2012). Measures of centrality based on the spectrum of the Laplacian. Phys. Rev. E.

[21] McInnes L., Healy J., Melville J. (2018). UMAP: Uniform Manifold Approximation and Projection for Dimension Reduction. arXiv.

[22] Pagnozzi A.M., Conti E., Calderoni S., Fripp J., Rose S.E. (2018). A systematic review of structural MRI biomarkers in autism spectrum disorder: A machine learning perspective. Int. J. Dev. Neurosci..

[23] Schmaal L., Ching C.R., McMahon A.B., Jahanshad N., Thompson P.M. (2020). Neuroimaging, genetics, and personalized psychiatry: Developments and opportunities from the ENIGMA consortium. Personalized Psychiatry.

[24] Button K.S., Ioannidis J.P., Mokrysz C., Nosek B.A., Flint J., Robinson E.S., Munafò M.R. (2013). Power failure: Why small sample size undermines the reliability of neuroscience. Nat. Rev. Neurosci..

[25] Iacoboni M., Dapretto M. (2006). The mirror neuron system and the consequences of its dysfunction. Nat. Rev. Neurosci..

[26] Beer J.S., John O.P., Scabini D., Knight R.T. (2006). Orbitofrontal cortex and social behavior: Integrating self-monitoring and emotion-cognition interactions. J. Cogn. Neurosci..

[27] Hardan A.Y., Girgis R.R., Lacerda A.L., Yorbik O., Kilpatrick M., Keshavan M.S., Minshew N.J. (2006). Magnetic resonance imaging study of the orbitofrontal cortex in autism. J. Child Neurol..

[28] Hill E.L. (2004). Executive dysfunction in autism. Trends Cogn. Sci..

[29] Cai R.Y., Richdale A.L., Uljarević M., Dissanayake C., Samson A.C. (2018). Emotion regulation in autism spectrum disorder: Where we are and where we need to go. Autism Res..

[30] Dubbelink L.M.O., Geurts H.M. (2017). Planning skills in autism spectrum disorder across the lifespan: A meta-analysis and meta-regression. J. Autism Dev. Disord..

[31] Leung R.C., Zakzanis K.K. (2014). Brief report: Cognitive flexibility in autism spectrum disorders: A quantitative review. J. Autism Dev. Disord..

[32] Bishop D.V., Norbury C.F. (2005). Executive functions in children with communication impairments, in relation to autistic symptomatology: I: Generativity. Autism.

[33] Barendse E.M., Hendriks M.P., Jansen J.F., Backes W.H., Hofman P.A., Thoonen G., Kessels R.P., Aldenkamp A.P. (2013). Working memory deficits in high-functioning adolescents with autism spectrum disorders: Neuropsychological and neuroimaging correlates. J. Neurodev. Disord..

[34] Solomon M., Ozonoff S.J., Ursu S., Ravizza S., Cummings N., Ly S., Carter C.S. (2009). The neural substrates of cognitive control deficits in autism spectrum disorders. Neuropsychologia.

[35] Amodio D.M., Frith C.D. (2006). Meeting of minds: The medial frontal cortex and social cognition. Nat. Rev. Neurosci..

[36] Xu J., Wang C., Xu Z., Li T., Chen F., Chen K., Gao J., Wang J., Hu Q. (2020). Specific functional connectivity patterns of middle temporal gyrus subregions in children and adults with autism spectrum disorder. Autism Res..

[37] Narzisi A., Muratori F., Calderoni S., Fabbro F., Urgesi C. (2013). Neuropsychological profile in high functioning autism spectrum disorders. J. Autism Dev. Disord..

[38] Southwick J.S., Bigler E.D., Froehlich A., DuBray M.B., Alexander A.L., Lange N., Lainhart J.E. (2011). Memory functioning in children and adolescents with autism. Neuropsychology.

[39] Van Rooij D., Anagnostou E., Arango C., Auzias G., Behrmann M., Busatto G.F., Calderoni S., Daly E., Deruelle C., Di Martino A. (2018). Cortical and subcortical brain morphometry differences between patients with autism spectrum disorder and healthy individuals across the lifespan: Results from the ENIGMA ASD Working Group. Am. J. Psychiatry.

[40] Rizzolatti G., Fabbri-Destro M. (2010). Mirror neurons: From discovery to autism. Exp. Brain Res..

[41] Carper R.A., Moses P., Tigue Z.D., Courchesne E. (2002). Cerebral lobes in autism: Early hyperplasia and abnormal age effects. Neuroimage.

[42] Jou R.J., Minshew N.J., Keshavan M.S., Hardan A.Y. (2010). Cortical gyrification in autistic and Asperger disorders: A preliminary magnetic resonance imaging study. J. Child Neurol..

[43] Travers B.G., Kana R.K., Klinger L.G., Klein C.L., Klinger M.R. (2015). Motor learning in individuals with autism spectrum disorder: Activation in superior parietal lobule related to learning and repetitive behaviors. Autism Res..

[44] Freitag C.M., Konrad C., Häberlen M., Kleser C., von Gontard A., Reith W., Troje N.F., Krick C. (2008). Perception of biological motion in autism spectrum disorders. Neuropsychologia.

[45] Cavanna A.E., Trimble M.R. (2006). The precuneus: A review of its functional anatomy and behavioural correlates. Brain.

[46] Schilbach L., Wohlschlaeger A.M., Kraemer N.C., Newen A., Shah N.J., Fink G.R., Vogeley K. (2006). Being with virtual others: Neural correlates of social interaction. Neuropsychologia.

[47] Cheng W., Rolls E.T., Gu H., Zhang J., Feng J. (2015). Autism: Reduced connectivity between cortical areas involved in face expression, theory of mind, and the sense of self. Brain.

[48] Lombardo M.V., Chakrabarti B., Bullmore E.T., Sadek S.A., Pasco G., Wheelwright S.J., Suckling J., Consortium M.A., Baron-Cohen S. (2010). Atypical neural self-representation in autism. Brain.

[49] Di Martino A., Ross K., Uddin L.Q., Sklar A.B., Castellanos F.X., Milham M.P. (2009). Functional brain correlates of social and nonsocial processes in autism spectrum disorders: An activation likelihood estimation meta-analysis. Biol. Psychiatry.

[50] Dickstein D.P., Pescosolido M.F., Reidy B.L., Galvan T., Kim K.L., Seymour K.E., Laird A.R., Di Martino A., Barrett R.P. (2013). Developmental meta-analysis of the functional neural correlates of autism spectrum disorders. J. Am. Acad. Child Adolesc. Psychiatry.

[51] Di Martino A., O’connor D., Chen B., Alaerts K., Anderson J.S., Assaf M., Balsters J.H., Baxter L., Beggiato A., Bernaerts S. (2017). Enhancing studies of the connectome in autism using the autism brain imaging data exchange II. Sci. Data.

[52] Uddin L.Q., Menon V. (2009). The anterior insula in autism: Under-connected and under-examined. Neurosci. Biobehav. Rev..

[53] Schaaf R.C., Toth-Cohen S., Johnson S.L., Outten G., Benevides T.W. (2011). The everyday routines of families of children with autism: Examining the impact of sensory processing difficulties on the family. Autism.

[54] Hyde K.L., Samson F., Evans A.C., Mottron L. (2010). Neuroanatomical differences in brain areas implicated in perceptual and other core features of autism revealed by cortical thickness analysis and voxel-based morphometry. Hum. Brain Mapp..

[55] Scheel C., Rotarska-Jagiela A., Schilbach L., Lehnhardt F.G., Krug B., Vogeley K., Tepest R. (2011). Imaging derived cortical thickness reduction in high-functioning autism: Key regions and temporal slope. Neuroimage.

[56] Grill-Spector K., Kourtzi Z., Kanwisher N. (2001). The lateral occipital complex and its role in object recognition. Vis. Res..

[57] Weigelt S., Koldewyn K., Kanwisher N. (2013). Face recognition deficits in autism spectrum disorders are both domain specific and process specific. PLoS ONE.

[58] Van der Hallen R., Evers K., Brewaeys K., Van den Noortgate W., Wagemans J. (2015). Global processing takes time: A meta-analysis on local-global visual processing in ASD. Psychol. Bull..

