# Supplementary material for: Node Centrality Measures Identify Relevant Structural MRI Features of Subjects with Autism
Source: Brain Sci. 2021 Apr 14;11(4):498. doi: 10.3390/brainsci11040498 (PMC8071038; doi:10.3390/brainsci11040498)
Supplement: Supplementary file 1 [file brainsci-11-00498-s001.pdf]

Supplementary Materials of the paper
Node Centrality Measures identify relevant structural MRI features of Subjects with Autism

Marcello Zanghieri [1], Giulia Menichetti [2], Alessandra Retico [3], Sara Calderoni [4], Gastone Castellani [5], Daniel Remondini [6]

[1] University of Bologna; marcello.zanghieri2@unibo.it
[2] Center for Complex Network Research, Department of Physics, Northeastern University, Boston, USA and Department of Medicine, Brigham and Women's Hospital, Harvard Medical School; g.menichetti@northeastern.edu
[3] National Institute for Nuclear Physics (INFN), Pisa Division; alessandra.retico@pi.infn.it
[4] Department of Developmental Neuroscience, IRCCS Stella Maris Foundation and Department of Clinical and Experimental Medicine, University of Pisa, Pisa, Italy; sara.calderoni@fsm.unipi.it
[5] University of Bologna; gastone.castellani@unibo.it
[6] University of Bologna; daniel.remondini@unibo.it
* Correspondence: gastone.castellani@unibo.it, daniel.remondini@unibo.it

S1. Stratification with respect to age and multi-site data collection

In this supplementary section, we show that the sMRI data of the subset of ABIDE-I subjects analyzed in this work (i.e., right-handed TD and AUT males, as explained in Section 2.1) is well mixed with respect to acquisition site and age, that thus we did not consider as confounding factors. Acquisition site and age were considered because they are the only variables reported for all subjects. Other information, such as Body Mass Index (BMI), is only reported for a minority of the subjects, making a detailed analysis impossible, thus we did not consider it.

The statistical tests on age distributions included in the main paper (Section 2.1, Section 3.1, and Table 1) were performed to show that selecting right-handed TD and AUT males introduces no age-related bias compared to the overall ABIDE-I dataset. The contribution of this additional analysis is checking that this restriction on subjects does not induce any artifact (such as clusters) of the sMRI brain features with respect to acquisition centers and age.

The following figures are visualizations of the selected subjects' brain features produced using *t*-SNE [1, 2] and UMAP [3], two established methods to embed high-dimensional data into a low-dimensional space to highlight possible stratifications or clusters. Datapoints (i.e. subjects) colored based on acquisition center or age. As it can be seen, no clusters or stratifications form. Therefore, it is possible to conclude that the cerebral data of the subset of ABIDE-I subjects considered in this study is well stratified as to both acquisition site and age.

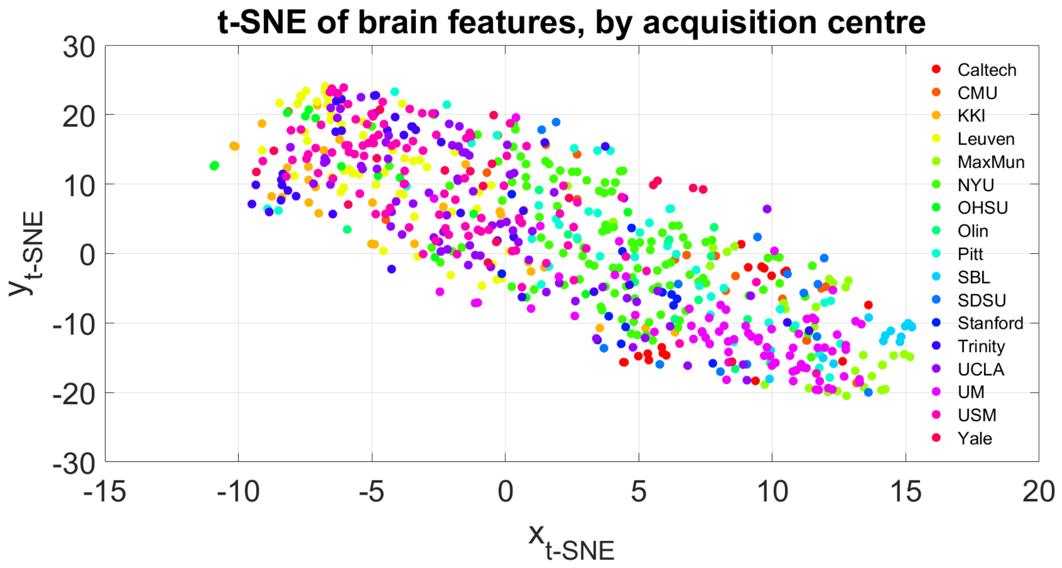
t-SNE of brain features, by acquisition centre
Caltech
CMU
KKI
Leuven
MaxMun
NYU
OHSU
Olin
Pitt
SBL
SDSU
Stanford
Trinity
UCLA
UM
USM
Yale
$y_{t\text{-SNE}}$
$x_{t\text{-SNE}}$
30
20
10
0
-10
-20
-30
-15
-10
-5
0
5
10
15
20

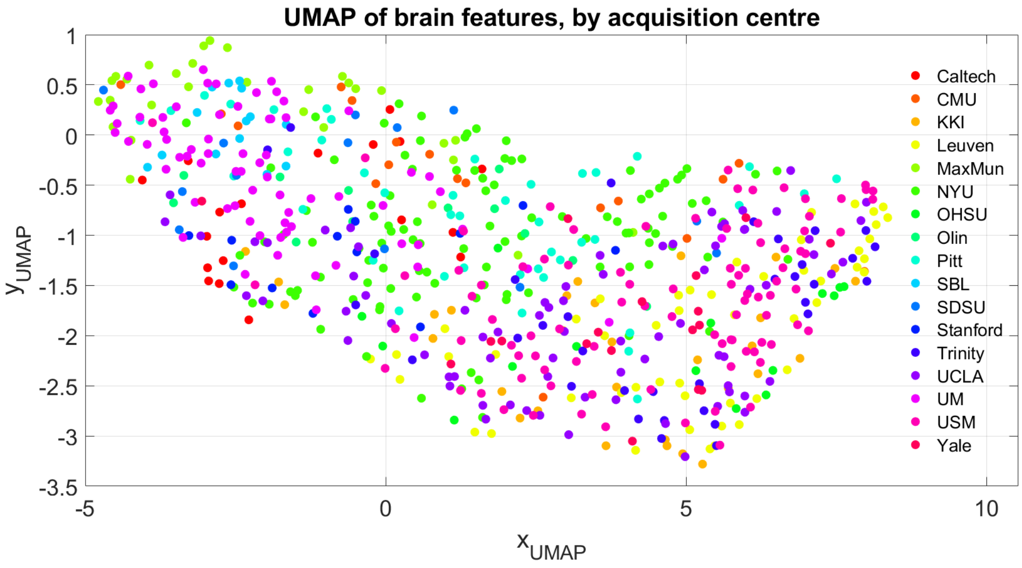
UMAP of brain features, by acquisition centre
Caltech
CMU
KKI
Leuven
MaxMun
NYU
OHSU
Olin
Pitt
SBL
SDSU
Stanford
Trinity
UCLA
UM
USM
Yale
y_{UMAP}
x_{UMAP}
1
0.5
0
-0.5
-1
-1.5
-2
-2.5
-3
-3.5
-5
0
5
10

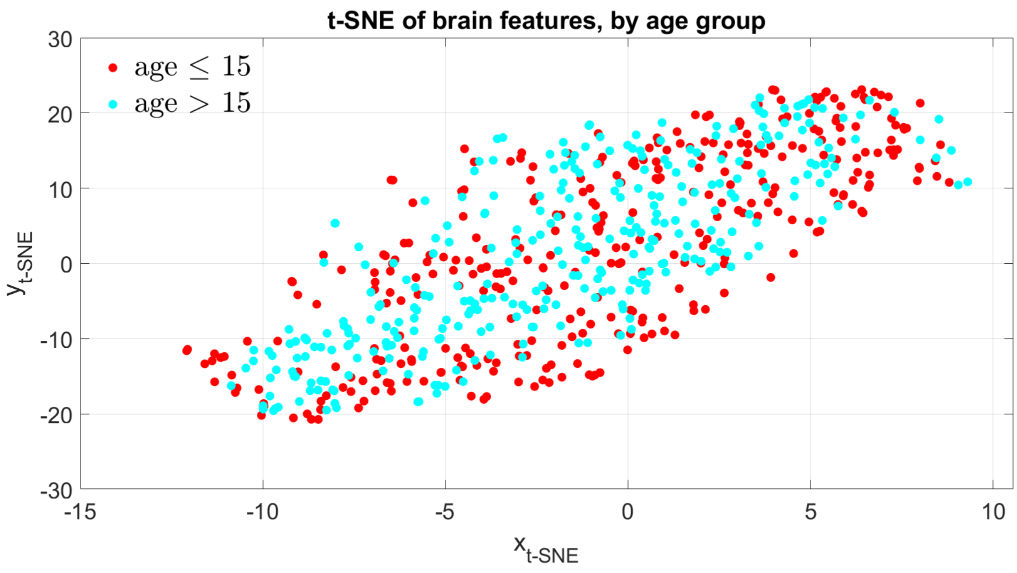
t-SNE of brain features, by age group
$\text{age} \leq 15$
$\text{age} > 15$
$y_{t\text{-SNE}}$
$x_{t\text{-SNE}}$
30
20
10
0
-10
-20
-30
-15
-10
-5
0
5
10

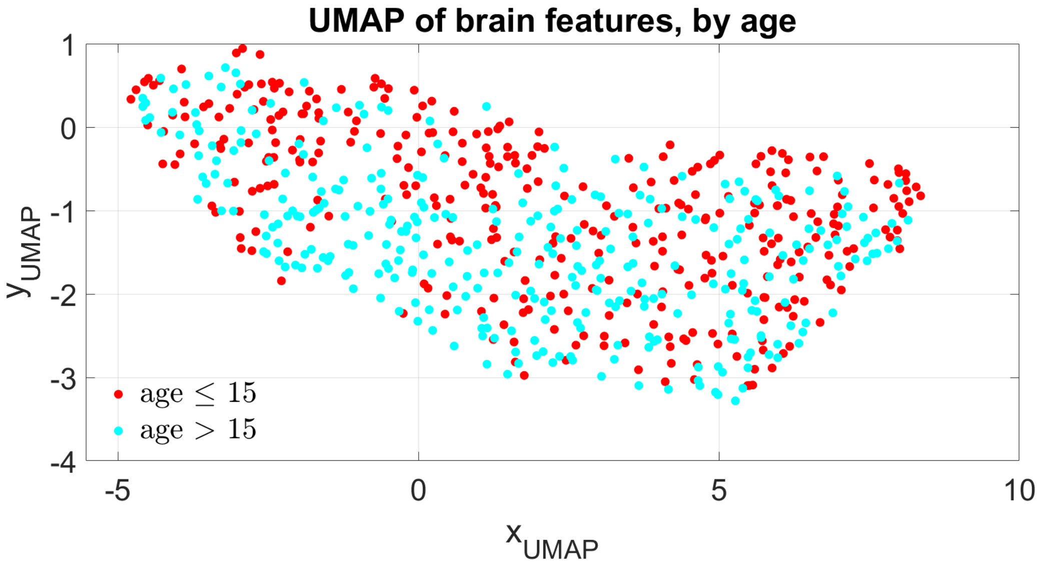

S2. Single feature statistical analysis

In this supplementary section, we provide more insight into the distributions of the single brain features of the ABIDE-I subjects considered in this study. All our work is based on second-order statistics (i.e. comparison of relationships between couples of features), but here we show the results of two statistical analyses performed at a single-feature level.

To compare control and AUT groups, we performed a parametric analysis (Student's T test) and a nonparametric test (Wilcoxon's ranksum test). We show here the results for the most significant features (P<0.05 after Bonferroni's post-hoc correction of statistical significance): we found 6 features significantly different for the parametric test, and 4 features for the nonparametric test, with 4 overlapping features in both tests.

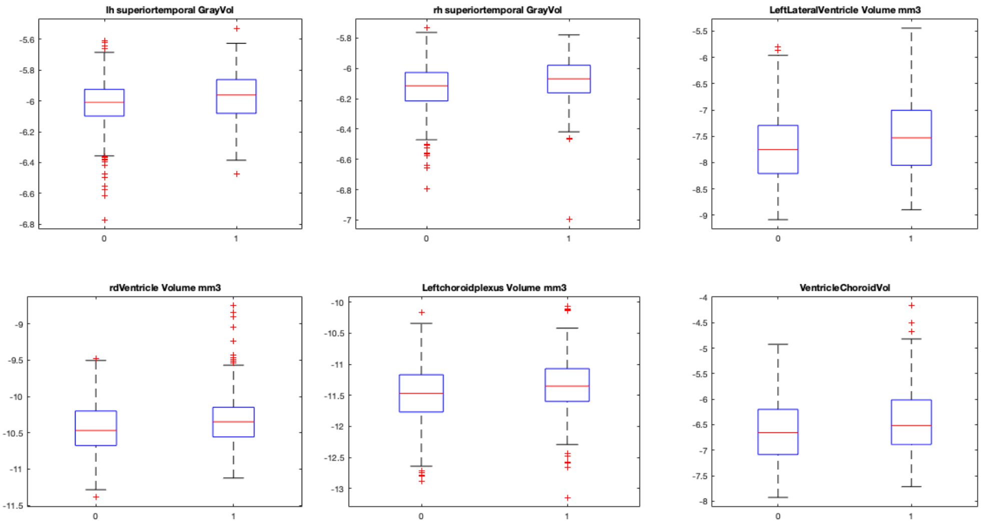
lh superiortemporal GrayVol
rh superiortemporal GrayVol
LeftLateralVentricle Volume mm3
rdVentricle Volume mm3
Leftchoroidplexus Volume mm3
VentricleChoroidVol

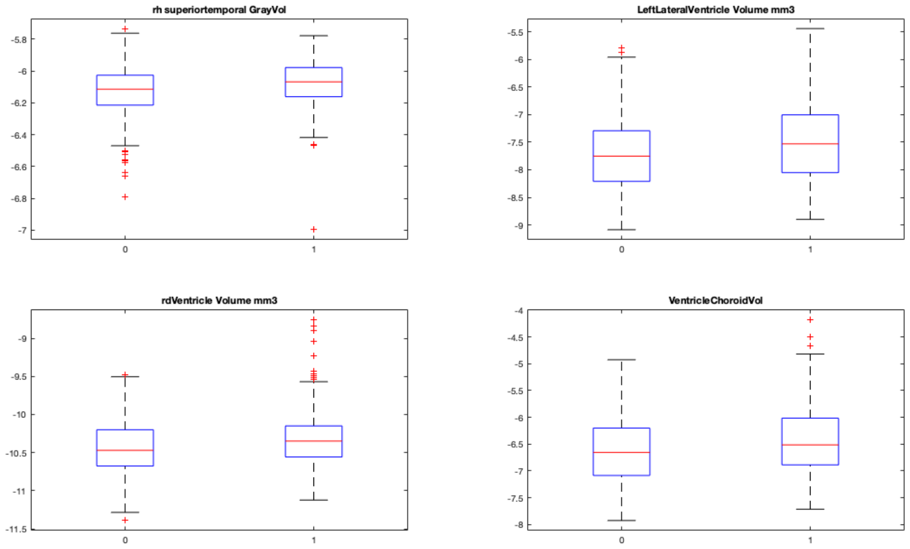
rh superiortemporal GrayVol
LeftLateralVentricle Volume mm3
rdVentricle Volume mm3
VentricleChoroidVol

S3. Results with resampling for both TD and AUT

To test the robustness of our analysis, we repeated the whole-network comparison stage of our pipeline performing the resampling procedure on both TD and AUT subject groups. This introduces variance for the AUT measures too, thus putting effect size and statistical significance to the test.

Specifically,

- for the TD case, we generate K_{suppl} = 1000 networks, using K_{suppl} random subsamples of M_{suppl} = 188 TD subjects;
- for the AUT case, we do the same (1000 networks, 188 samples for each resampling).

Apart from the resampling procedure, all the other steps of the analysis (including visualization) are kept identical to the main experiment, as explained in Section 3. The results are displayed in the figures and in the table below. First, the two cluster maps (left: TD; right: AUT) show that the centrality measures form the same 4 clusters as for the resampling procedure described in the paper. Secondly, the distributions of the whole-network average of every centrality show the same behavior as in the main experiment: the distributions are clearly separated, and the differences have the same sign as in the main experiment.

The reported table is analogous to Table 2 of the main paper, and reports the *z*-score (i.e, difference of the means, normalized by the pooled variance) for the twelve centrality measures considered: we remark that the top centrality measure for each measure cluster is the same as in the main experiment: Betweenness, Clustering Coefficient, and Weighted Spectral Centrality are the top-separating features inside Cluster I, II, and IV, respectively, whereas the Inverse Participation Ratio constitutes a cluster on its own, as in the main experiment.

Thus, these supplementary results confirm the robustness of our network-based approach, independently of the subsampling strategy adopted.

Cluster	Centrality measure	*z*-score
I	betweenness	1.66
	weighted betweenness	1.65
II	clustering coefficient	2.11
	nn-strength	1.73
	nn-degree	1.70
	closeness	-1.69
	strength	1.66
	weighted closeness	-1.02
	degree	0 by constr.
III	inverse participation ratio	1.33
IV	weighted spectral	-2.94
	spectral	-2.61

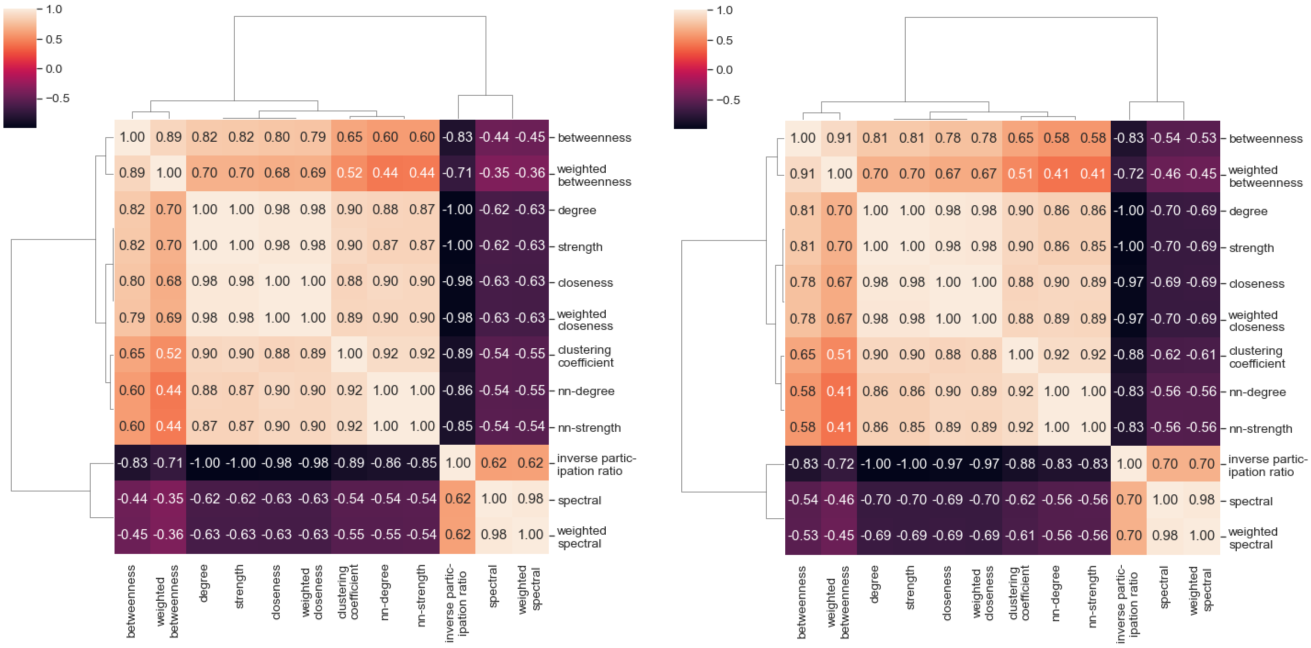

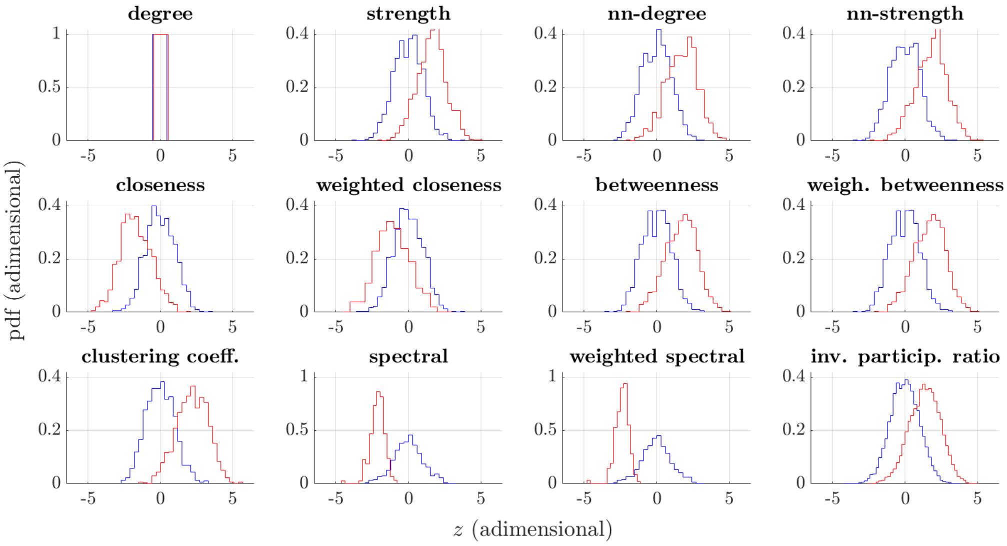

References

[1] Visualizing High-Dimensional Data Using *t*-SNE, van der Maaten, L.J.P., Hinton, G.; Journal of Machine Learning Research, 2008.

[2] Accelerating *t*-SNE using Tree-Based Algorithms, van der Maaten, L.J.P.; Journal of Machine Learning Research, 2014.

[3] UMAP: Uniform Manifold Approximation and Projection for Dimension Reduction, McInnes, L., Healy, J.; 2018.
